# Assessment of Undernutrition Among Children in 55 Low- and Middle-Income Countries Using Dietary and Anthropometric Measures

**DOI:** 10.1001/jamanetworkopen.2021.20627

**Published:** 2021-08-12

**Authors:** Markus Heemann, Rockli Kim, Sebastian Vollmer, S. V. Subramanian

**Affiliations:** 1 Department of Development Economics, Centre for Modern Indian Studies, University of Goettingen, Göttingen, Germany; 2 Division of Health Policy and Management, Korea University College of Health Science, Seoul, South Korea; 3 Interdisciplinary Program in Precision Public Health, Department of Public Health Sciences, Graduate School of Korea University, Seoul, South Korea; 4 Harvard Center for Population and Development Studies, Cambridge, Massachusetts; 5 Department of Social and Behavioral Sciences, Harvard T.H. Chan School of Public Health, Boston, Massachusetts

## Abstract

**Importance:**

Evidence on the suitability of anthropometric failure (ie, stunting, underweight, and wasting) as a stand-alone measure of child undernutrition can inform global and national nutrition and health agendas.

**Objective:**

To provide a comprehensive estimate of the prevalence of child undernutrition by evaluating both dietary and anthropometric measures simultaneously across 55 low- and middle-income countries.

**Design, Setting, and Participants:**

This was a cross-sectional study that used Demographic and Health Surveys program data from July 2009 to January 2019, to allocate children into dietary and anthropometric failure categories. Nationally representative household surveys were conducted in 55 low- and middle-income countries. Participants included children aged 6 to 23 months who were born singleton and had valid anthropometric measures as well as available 24-hour food intake recollection. Data analysis was conducted from August 23 to October 22, 2020.

**Exposures:**

Two factors were considered to allocate children into the respective categories. Dietary failure was based on the World Health Organization standards for minimum dietary diversity. Anthropometric failure was constructed using the World Health Organization child growth reference standard *z* score for stunted growth, muscle wasting, and less than average weight for age.

**Main Outcomes and Measures:**

Dietary and anthropometric failures were cross-tabulated, which yielded 4 potential outcomes: dietary failure only, anthropometric failure only, both failures, and neither failure. Total child populations for each category were extrapolated from United Nations population estimates.

**Results:**

Of the 162 589 children (median age [range], 14 months [6-23 months]; 83 467 boys [51.3%]; 78 894 Asian children [48.5%]) in our sample, 42.9% of children had dietary failure according to the standard World Health Organization definition without being identified as having anthropometric failures. In all, 34.7% had both failures, 42.9% had dietary failure only, 8.3% had anthropometric failure only, and 14.1% had neither failure. Dietary and anthropometric measures were discordant for 51.2% of children; these children had nutritional needs identified by only 1 of the 2 measures. Dietary failure doubled the proportion of children in need of dietary interventions compared with anthropometry alone (43%). A total of 45.3 million additional children who experienced undernutrition in these 55 countries were not captured through the evaluation of anthropometric failures only. These results were consistent across geographic regions.

**Conclusions and Relevance:**

The results of this cross-sectional study suggest that the current standard of measuring child undernutrition by estimating the prevalence of anthropometric failure should be complemented with dietary and food-based measures. Anthropometry alone may fail to identify many children who have insufficient dietary intake.

## Key Points

**Question:**

Is anthropometric failure an adequate stand-alone measure to estimate global child undernutrition, and how do estimates change when dietary measures are also taken into account?

**Findings:**

In this cross-sectional study of 162 589 children (aged 6-23 months) in 55 low- and middle-income countries, dietary and anthropometric measures were discordant for 51% of children. A total of 43% of children (equivalent to 45.3 million children) had dietary failure without showing any signs of anthropometric failures.

**Meaning:**

The findings of this study suggest that the current standard of measuring child undernutrition with anthropometric failure should be complemented with diet- and food-based measures.

## Teaser

This cross-sectional study assesses the prevalence of child undernutrition through an evaluation of dietary and anthropometric measures in 55 low- and middle-income countries.

## Introduction

Child undernutrition is a significant burden across the globe, with World Health Organization (WHO) estimates reporting more than 205 million children who are undernourished, particularly in low- and middle-income countries (LMICs).^1^ Undernutrition in early childhood has been linked to significant harm in physical as well as cognitive development.^2,3,4,5^ As such, preventing and treating child undernutrition is not only relevant to achieve the Sustainable Development Goal No. 3 “Good Health and Well-being,” which is part of the United Nations’ 2030 Agenda for Sustainable Development, but also to address root causes of health inequality.

There are 2 relevant measures regarding the evaluation of children’s nutrition: anthropometry and diet. Although both measures are equally relevant, scientific research and policy agenda most often rely on anthropometry (the study of measurements or proportions of the human body), more specifically, anthropometric failures, when assessing the degree and magnitude of undernourishment among children.^6,7^ A child is considered to have anthropometric failure if they have stunted growth, are underweight, have muscle wasting, or have a combination of these 3 descriptions. Anthropometric failure is an important measure that is closely related to food and often leads to targeted nutrition-based interventions.^8,9^ At the same time, it is a fairly complex indicator capturing genetic, environmental and household factors as well.^10^ This means that anthropometric failure may occur even when nutritional intake is generally sufficient. Similarly, not all nutritional deficiencies would be expected to result in anthropometric failure.^11^ Thus, although anthropometric failure may indicate undernutrition, it is an imprecise measure for assessing the true extent of undernutrition burden and for identifying precise target groups for nutrition interventions.^12,13,14,15^

To examine the association between the prevalence of diet and anthropometric failure, Beckerman-Hsu et al^13^ recently established a typology framework consisting of 4 dietary and anthropometric failure (DAF) categories: dietary failure only (DFO), anthropometric failure only (AFO), both failures (BF), and neither failure (NF) (Table 1). Using Indian Demographic Health Survey (DHS) program data to assign children into these 4 categories, they found that 36.3% of children had micronutrient deficiency without showing any sign of anthropometric failure. Including those children who also showed signs of anthropometric failure, more than 80.3% of children did not meet the WHO standard for minimum acceptable diet (compared with 53.8% who showed signs of anthropometric failure). By using this newly proposed typology, that study^13^ showed that considering anthropometric measures only to estimate the extent of undernutrition does not capture the full burden and that many children with insufficient micronutrient intake remain “hidden,” which leads to imprecise target groups for nutrition interventions. Our analyses for India showed very similar results (Table 1). Those findings also have important implications for policymakers, for example, when allocating budgets for targeted interventions.

**Table 1.** Dietary and Anthropometric Failure (DAF) Categories^a^

DAF category	Sample size India	% Population India (95% CI)
Both failures	28 867	44.9 (44.5-45.3)
Dietary failure only	23 906	35.8 (35.4-36.2)
Anthropometric failure only	6294	9.9 (9.7-10.1)
Neither failure	6430	9.4 (9.2-9.6)

^a^ The categories were established by Beckerman-Hsu et al,^13^ who used Indian Demographic Health Survey program data to assign children to the 4 categories.

Given that the study by Beckerman-Hsu et al^13^ was restricted to India, there is a need to apply the DAF framework to a larger cross-country sample. If the results presented for India can be validated for a global sample, it becomes clear that measures of diet and micronutrient intake need to be much more prevalent in future research in childhood undernutrition. Applying the DAF globally has 2 potential benefits: (1) the identification of children with nutritional need who were previously undetected, giving more precise target groups and estimates of the true extent of child undernutrition, and (2) the potential identification of the precise food-based needs (ie, which micronutrients are missing) that would enable policymakers to make evidence-based prioritization for resource allocation and to monitor the progress of respective interventions at both national and global levels.

In this study, we extended the DAF framework introduced by Beckerman-Hsu et al^13^ to 55 LMICs for which DHS data were available. We calculated country-specific typology patterns and derived an estimate of the magnitude of the true burden caused by anthropometric failure and micronutrient deficiency together among children aged 6 to 23 months. We also estimated how many children may be overlooked if the global health community continues to assess child undernutrition based on anthropometric failure alone.

## Methods

### Data

The DHS program conducts survey waves on population, health, and nutrition for nationally representative samples across the globe. This cross-sectional analysis was reviewed by the Harvard T.H. Chan School of Public Health Institutional Review Board and was considered exempt from full review because the study was based on an anonymous public use data set with no identifiable information on the survey participants. Households were chosen in a 2-stage process that included a selection of enumeration areas and then a sample of households for each enumeration area. Verbal informed consent was sought from respondents by reading a prescribed statement to the respondent and recording in the questionnaire whether the respondent consented. This study followed the Strengthening the Reporting of Observational Studies in Epidemiology (STROBE) reporting guideline for cross-sectional studies. For each country, we used data from the most recent DHS survey wave, which was conducted after July 2009, to January 2019, that contained information on children’s dietary intake and height and weight measurements as well as month-specific age information. These surveys were available for 55 countries. For Colombia, we used the DHS wave 6 data from 2010 instead of wave 7 data from 2015, as it contained many more data points on nutrition relevant to our analyses. We also used population data drawn from the United Nations World Population Prospect (2018)^16^ to estimate the total child population count corresponding to each DAF type. As such, the 7 largest countries that considered children aged 6 to 23 months (ie, India, Nigeria, Pakistan, Democratic Republic of Congo, Bangladesh, Ethiopia, and Egypt) contributed approximately  66% of the total sample of 55 countries. Mean per capita income information was drawn from PovCalNet (an online analysis tool for monitoring global rates of resource-constrained regions), the United Nations University World Institute for Development Economics Research, and Human Development Reports.

### Study Population and Sample Size

Our analyses included children aged 6 to 23 months, which is in line with the WHO Indicators for assessing infant and young feeding practices.^17^ We obtained nationally representative DHS data from 55 LMICs for 208 044 children aged 6 to 23 months who were the youngest child in their household and who lived together with their mother.^18^ Of our sample of 208 020 children, 166 929 had height and weight measurements used to derive the *z* scores to identify anthropometric failure. Of these children, 162 589 had dietary data, yielding our final sample size of 162 589 children. Of the final sample size, 83 467 (51.3%) were boys, 79 122 (48.7%) were girls, 56 880 (35.0%) were between 6 and 11 months old, and 105 708 (65.0%) were between 12 and 23 months old (eFigure 1 in the Supplement).

### Outcomes

#### Anthropometric Failure

Children’s anthropometry data (measured height and weight) were obtained from the DHS data. The WHO child growth reference standard *z* score was used to identify children with stunted growth (height-for-age *z* score <2), who were underweight (weight-for-age *z* score <2), and had muscle wasting (weight-for-height *z* score <2). Anthropometric failure was defined as a binary variable with anthropometric failure prevailing if a child had stunted growth, was underweight, had muscle wasting, or a combination of these failures.

#### Dietary Failure

Children’s dietary data were based on a 24-hour recall in the DHS surveys. Children’s consumption of the following 8 food groups was collected: (1) grains, roots, and tubers; (2) legumes and nuts; (3) dairy products (milk, yogurt, cheese); (4) flesh foods (meat, fish, poultry, and liver/organ meats); (5) eggs; (6) vitamin A–rich fruits; (7) vegetables; and (8) breast milk. We defined dietary failure as a binary variable, assigning the outcome *yes* if the child’s dietary intake did not meet the minimum dietary requirements as defined by the WHO classification from 2017, which required an intake of a minimum of 5 of 8 different food categories.^17^ As recommended by the DHS,^18^ food categories with answers such as “do not know” or that were missing individual data points were assigned an outcome of *no*. Given our sample of approximately 200 000 responses for each food category and approximately 350 responses for each category being either do not know or missing data points, we do not believe that assigning the missing 0.2% to the no category significantly changed our results. The indicator for minimum dietary diversity was created as a way to use dietary data to capture the micronutrient density of the diets in children aged 6 to 23 months and has been validated previously.^19,20^ Therefore, children who did not reach minimum dietary diversity were considered to have unmet nutritional need regardless of prevailing anthropometric failure.

### Statistical Analysis

We cross-tabulated anthropometric and dietary failures yielding 4 potential outcomes: DFO, AFO, BF, and NF. We calculated the prevalence of DAF. For each respective DAF category, we calculated the prevalence of DAF on national levels for all 55 LMICs. Additionally, we estimated the burden, in terms of total number of children, of each DAF category by using United Nations population data from 2018 for children aged 0 to 5 years for these 55 countries. Given that DHS is a nationally representative data set, we extrapolated the share of children aged 6 to 23 months (30.3%) from children aged 0 to 5 years (353.0 million) to the national population estimates according to the United Nations, yielding our final population size (105.8 million) of children aged 6 to 23 months for the 55 countries included in our analysis. We considered 5% levels of significance, and hypothesis tests were 2-sided. All analyses were conducted on Stata, version 16.0 (StataCorp). These data were analyzed from August 23 to October 22, 2020.

## Results

### Overall Child Undernutrition Using DAF Categories

This study included a total of 162 589 children (median age [range], 14 months [6-23 months]; 83 467 boys [51.3%]; 78 894 Asian children [48.5%]). Across all 55 LMICs and weighted by country size, 77.6% of the total sample of children aged 6 to 23 months were shown to have dietary failure, whereas only 43.0% had at least 1 form of anthropometric failure. The most common category was DFO in 42.9% of children, followed by BF in 34.7%, NF in 14.1%, and AFO in 8.3% (Table 2). Dietary and anthropometric measures were discordant for 51.2% of children; these children had nutritional needs identified by only 1 of the 2 measures (DFO + AFO). Although these results were strongly influenced by India, which accounted for roughly one-third of the total child population in our final sample, the results did not change significantly for DFO when we considered unweighted averages (DFO, 45.9%; BF, 26.4%; NF, 19.7%; AFO, 8.1%) (eTable 1 in the Supplement).

**Table 2.** Estimated Prevalence of Children Within DAF Categories Among 162 589 Children Aged 6-23 Months in 55 Countries

DAF category	Sample size	Estimated child population (million)	% (95% CI)^a^
Both failures	55 194	36.7	34.7 (34.5-35.0)
Dietary failure only	67 670	45.3	42.9 (42.6-43.1)
Anthropometric failure only	14 432	8.8	8.3 (8.2-8.4)
Neither failure	25 293	14.9	14.1 (14.0-14.3)
Total	162 589	105.8	100 (NA)

Abbreviations: DAF, dietary anthropometric failure; NA, not applicable.

^a^ Percentages are weighted by country’s child population.

Using the weighted prevalence of DAF in Table 2, we estimated the total number of children in different categories of DAF and found that DFO was the largest category, with an estimated population of 45.3 million children, followed by BF with 36.7 million children, NF with 14.9 million children, and AFO with 8.8 million children (Table 2). A more granular look at the prevalence of dietary failure and the prevalence of individual causes of anthropometric failure (ie, stunted growth, muscle wasting, and lower-than-average weight) can be found in eTable 2 in the Supplement.

### Country-Specific Analysis of DAF

Table 3 shows country-specific estimates for the prevalence of the DAF categories together with estimated population numbers. A certain degree of variation in the share of each DAF category was found for different countries. Although the Maldives (51.5%) and Peru (57.2%) had fairly large shares of children with NF, Niger (53.6%), Burkina Faso (47.9%), and Burundi (47.6%) had BF for roughly half of their child population aged 6 to 23 months. Gabon (66.2%), Haiti (62.6%), and Liberia (60.9%) had large shares of children in the DFO category, which captures children with nutritional needs who are missed when only anthropometric measures are evaluated. For 41 of the 55 LMICs, DFO was the largest category. For 38 LMICs, at least 40% of children were in the DFO category, further highlighting the importance of capturing nutritional intake in addition to anthropometric failure. In terms of the total number of children with DFO, the largest 8 countries contributed approximately 66.7% to the total number of children with DFO. Of those, India was the largest, contributing 28.0% to the total DFO number (Table 3).

**Table 3.** Share and Estimated Child Population for Each of the DAF Categories Across 55 Countries

Country	No. of children based on total population, ×10^3^					DAF category share, %				Proportion of total DFO burden, %
	BF	DFO	AFO	NF	Total	BF	DFO	AFO	NF	
India	15 938	12 706	3507	3338	35 489	44.9	35.8	9.9	9.4	28.0
Nigeria	2897	3949	687	1423	8957	32.3	44.1	7.7	15.9	8.7
Pakistan	2216	3856	341	842	7254	30.5	53.2	4.7	11.6	8.5
Congo Democratic Republic	1684	2239	280	488	4690	35.9	47.7	6.0	10.4	4.9
Ethiopia	1758	2238	175	398	4568	38.5	49.0	3.8	8.7	4.9
Egypt	795	1958	459	1048	4259	18.7	46.0	10.8	24.6	4.3
Bangladesh	1597	1826	468	780	4671	34.2	39.1	10.0	16.7	4.0
Tanzania	838	1438	197	431	2903	28.9	49.5	6.8	14.8	3.2
Uganda	570	1086	156	378	2190	26.0	49.6	7.1	17.2	2.4
Kenya	423	964	212	579	2177	19.4	44.3	9.7	26.6	2.1
Cote d’Ivoire	447	800	46	63	1355	33.0	59.0	3.4	4.6	1.8
Myanmar	315	781	81	209	1385	22.7	56.4	5.8	15.1	1.7
Ghana	221	778	84	235	1318	16.8	59.1	6.4	17.8	1.7
South Africa	353	759	138	379	1629	21.7	46.6	8.5	23.3	1.7
Cameroon	361	653	91	277	1381	26.2	47.2	6.6	20.0	1.4
Mozambique	556	644	242	239	1681	33.1	38.3	14.4	14.2	1.4
Angola	511	644	187	292	1634	31.3	39.4	11.4	17.9	1.4
Mali	286	509	75	143	1013	28.3	50.2	7.4	14.1	1.1
Niger	684	490	40	60	1275	53.6	38.5	3.2	4.7	1.1
Burkina Faso	488	481	15	35	1019	47.9	47.2	1.5	3.4	1.1
Colombia	74	453	86	523	1136	6.5	39.9	7.5	46.0	1.0
Senegal	162	444	32	127	764	21.3	58.0	4.1	16.6	1.0
Chad	322	425	39	33	819	39.3	51.9	4.8	4.1	0.9
Yemen	542	420	123	149	1234	43.9	34.0	10.0	12.1	0.9
Malawi	224	406	57	116	803	27.9	50.6	7.1	14.4	0.9
Zambia	265	368	63	125	821	32.3	44.8	7.7	15.3	0.8
Zimbabwe	162	318	38	94	612	26.4	51.9	6.2	15.4	0.7
Benin	134	273	51	88	546	24.6	50.1	9.3	16.1	0.6
Guinea	164	259	28	56	507	32.3	51.2	5.5	11.0	0.6
Nepal	213	255	142	214	824	25.8	30.9	17.3	26.0	0.6
Rwanda	177	248	51	111	586	30.1	42.3	8.7	18.9	0.5
Tajikistan	72	248	17	75	411	17.4	60.2	4.2	18.2	0.5
Togo	96	232	24	51	403	23.8	57.6	6.0	12.6	0.5
Cambodia	122	213	80	154	567	21.4	37.5	14.0	27.1	0.5
Haiti	61	212	11	55	339	17.9	62.6	3.3	16.1	0.5
Burundi	284	212	49	52	598	47.6	35.5	8.2	8.8	0.5
Peru	66	194	98	478	835	7.9	23.2	11.8	57.2	0.4
Sierra Leone	138	176	26	29	369	37.3	47.8	7.0	7.8	0.4
Liberia	81	155	4	14	254	31.7	60.9	1.7	5.7	0.3
Congo	75	154	13	35	278	27.1	55.5	4.7	12.8	0.3
Dominican Republic	21	142	12	148	323	6.4	44.1	3.7	45.8	0.3
Kyrgyz Republic	30	138	15	82	265	11.3	52.2	5.5	31.0	0.3
Guatemala	127	137	154	200	618	20.5	22.1	25.0	32.4	0.3
Honduras	29	100	34	162	324	8.9	30.7	10.5	49.9	0.2
Gambia	41	75	5	11	132	31.4	56.7	3.9	8.0	0.2
Gabon	17	69	3	15	105	16.6	66.2	3.0	14.2	0.2
Namibia	29	60	4	24	117	24.8	51.3	3.2	20.6	0.1
Lesotho	26	50	3	9	87	29.6	57.2	3.3	9.9	0.1
Armenia	6	36	2	21	66	9.1	55.1	3.3	32.5	0.1
Albania	2	22	3	24	52	4.1	43.2	6.6	46.1	0.0
Comoros	13	18	5	5	39	32.1	44.8	11.6	11.5	0.0
Timor-Leste	23	13	9	5	50	46.5	25.5	18.0	10.0	0.0
Guyana	3	8	3	9	23	12.4	37.3	12.7	37.5	0.0
Sao Tome and Principe	3	3	2	3	10	25.9	27.4	19.5	27.1	0.0
Maldives	1	2	2	5	11	7.7	21.8	19.0	51.5	0.0
Total										
Weighted^a^	36 738	45 337	8769	14 934	105 778	34.7	42.9	8.3	14.1	100
Unweighted^b^	NA	NA	NA	NA	NA	26.4	45.9	8.1	19.7	NA

Abbreviations: AFO, anthropometric failure only; BF, both failures; DAF, dietary and anthropometric failure; DFO, dietary failure only; NA, not applicable; NF, neither failure.

^a^ Weighted by population weights and country size.

^b^ Weighted by population weights but not country size.

### DAF by Geographic Region and Country-Level Income

The level of a country’s share of children with DFO appeared to be consistent across different geographic regions and income levels. Although NF and BF shares varied widely, DFO accounted for most children in all geographic regions, ranging from approximately  35% of children in Central and South America to approximately  50% in Europe (ie, Albania and Armenia) (Table 4). Country-level income levels (measured as mean annual household income per capita) did not appear to substantially affect the share of children with DFO (Figure). Although the prevalences of BF and NF were significantly associated with a country’s income level, DFO was associated at a 10% level of significance, and AFO had no significant association. The distribution of DAF category shares for different mean income per capita levels is displayed in eFigure 2 in the Supplement.

**Table 4.** Share of 4 Dietary and Anthropometric Failure (DAF) Types Across Geographic Regions in Europe, Asia, Africa, and South America

Region	Asia	Africa	South America	Europe
DAF category share, % (95% CI)				
Both failures	40.4 (40.0-40.7)	30.6 (30.3-31.0)	10.5 (10.1-11.0)	6.9 (5.4-8.4)
Dietary failure only	39.2 (38.9-39.6)	47.2 (46.8-47.6)	34.6 (33.9-35.3)	49.9 (47.0-52.7)
Anthropometric failure only	9.2 (9.0-9.4)	7.2 (7.0-7.4)	11.1 (10.6-11.6)	4.8 (3.5-6.0)
Neither failure	11.2 (11.0-11.4)	15.0 (14.7-15.2)	43.8 (43.0-44.5)	38.5 (35.7.0-41.3)
No. of countries in sample	9	37	7	2
Sample size	78 894	65 483	17 059	1153
Total estimated child population (aged 6-23 mo)	52.1 million	50.0 million	3.6 million	117.3 thousand

**Figure.**
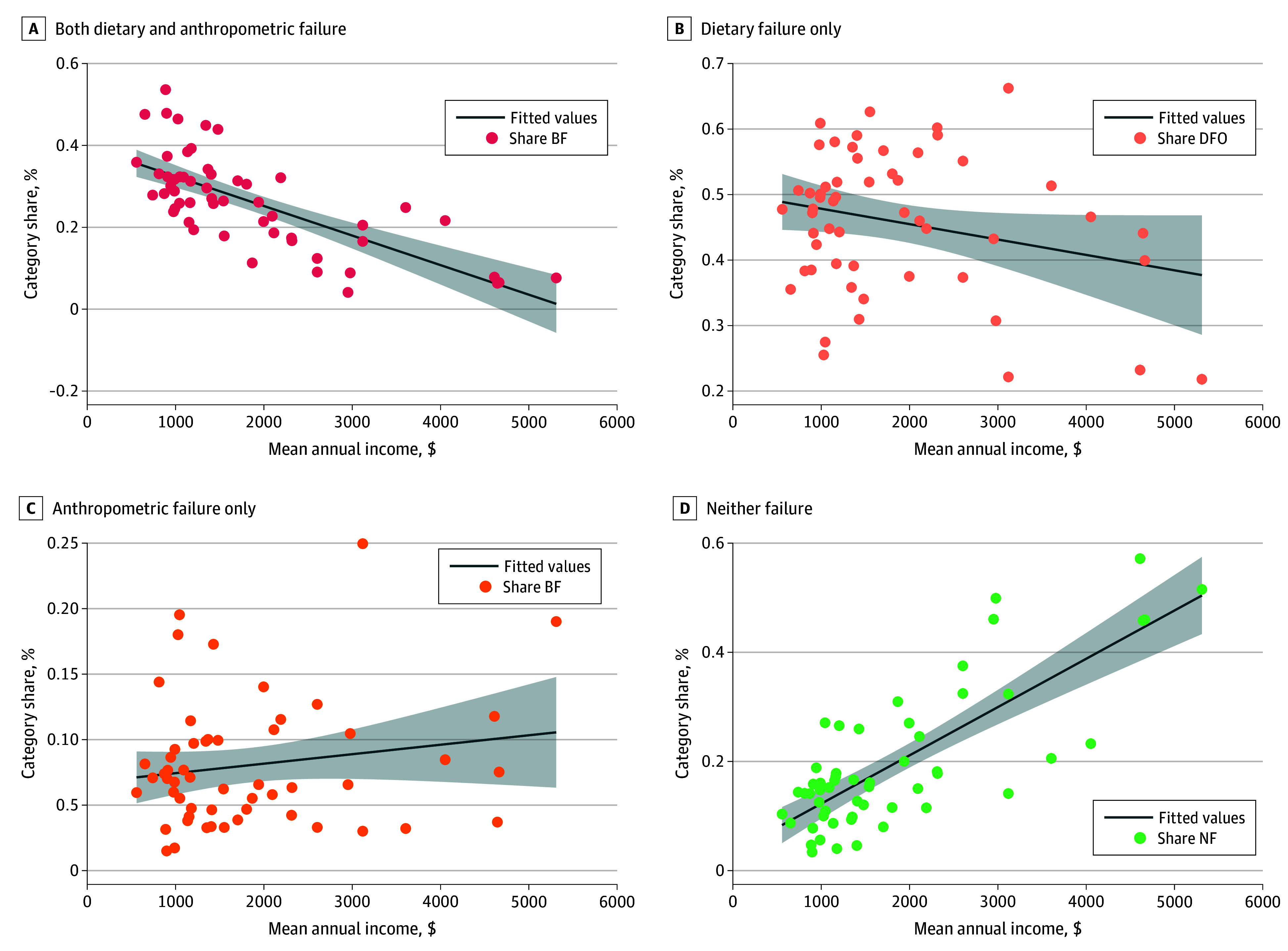
Correlation Between Country-Level Mean Income per Capita and Dietary and Anthropometric Failure (DAF) Category Share The DAF categories include (A) both failures (BF), (B) dietary failure only (DFO), (C) anthropometric failure only (AFO), and (D) neither failure (NF). The gray shaded areas indicate 95% CI. Income level was measured in US dollars.

### Variation Within DAF

It may take time for anthropometric failure to manifest in children; thus, children with BF may typically be older than children with DFO. At the same time, certain food groups may explain the allocation into the respective DAF categories, such that certain food groups may be responsible for DFO, AFO, and BF. Therefore we evaluated these 2 factors as a part of the robustness testing for this study.

eTable 3 in the Supplement shows the average age in months for each of the DAF categories across the region. The category AFO had the oldest average age in months across all regions, and DFO had the lowest average age in 3 of the 4 regions. However, when we compared the differences between DFO and BF (because both have dietary failure and thus are a better comparison than DFO and AFO), the variation was moderate: between −0.1 to 1.7 months of average age.

eFigures 3 and 4 in the Supplement present the variation of average consumption level of the previously mentioned 8 different food groups, both for the whole sample and for each region. Average consumption patterns for each of the food groups were similar for both categories without dietary failure (AFO and NF) as well as the categories with dietary failure (DFO and BF). This finding suggests that there was not a particular micronutrient that was responsible for the anthropometric failure. Average consumption levels were particularly low for legumes and nuts, flesh foods, eggs, and vegetables. Overall, although certain groups were more responsible for causing dietary failure, there was no indication that certain micronutrients cause anthropometric failure, which suggests that micronutrient intake should be examined next to anthropometric failure to capture nutrition deficiencies.

## Discussion

Our cross-sectional study found that approximately 4 of every 10 children in our analytic sample of 55 LMICs had no anthropometric failures but were identified as having dietary failure (the DFO category); this result included more than 45 million children aged 6 to 23 months with unmet dietary need who were not identified by measures focused solely on anthropometry. Approximately one-third of children had both dietary and anthropometric failures, and 4 of 5 children did not meet the minimum dietary diversity as recommended by WHO, which suggests the depth of nutritional need among a large proportion of the global child population in LMICs. Although there was some variation across countries, the relevance of the DFO category was consistent across different geographic regions and country-level mean income, as was within-category variation of age and food group consumption.

Future research will be needed to identify improved ways of measuring dietary intake, ideally over a longer period of time to account for the fact that the past 24 hours may not be reflective of regular daily alimentation. We used the WHO minimum dietary diversity indicator, but other dietary indicators may be considered, particularly those that combine the cost-effectiveness of a survey question (rather than relying on biomedical information and medical examination results) with increased reliability over time and sensitivity.^21^ Similarly, given varying degrees of nutritional intake at different stages in early childhood, other indicators may be considered for different age groups. Given that Beckerman-Hsu et al^13^ found larger variations of the DAF categories at the district level in India, future research must also take a more granular look into county- and district-level DAF prevalence to properly choose and prioritize food policy interventions.

The fact that DFO was found to be a primary category across geographic regions and country-level income suggests the need to consider dietary intake along with anthropometric failure when assessing the extent of nutritional burden in a more comprehensive manner and to successfully foster food security across the globe. We acknowledge that anthropometric failure is an important measure that is associated with nutrition and often leads to targeted interventions that include providing food to the children in need.^8,9^ We therefore do not intend to argue for a replacement or substitution of anthropometric failure in global health research and policy. Instead, our study findings suggest that the consideration of anthropometric failures alone may leave undetected large parts of the population with nutritional deficiencies. Across the 55 LMICs considered in our analysis, this translated to more than 45 million children who appeared to not have a nutritional deficiency based on anthropometry measures but in fact were in need of better nutrition. Analysis of both dietary intake and anthropometric failure may enable more precise identification of children with nutritional needs and may facilitate policymakers to develop more effective, equitable, and targeted interventions that could increase global food security.

### Limitations

Our study had 2 sources of data limitations. First, given that the dietary data were self-reported by mothers based on a 24-hour recall, the innate nature of the data was subject to some measurement error. However, DHS data on dietary intake have been found to be appropriate for the population level.^22^ Second, our estimates may have been biased by survey nonresponse and missing data for specific survey items or countries. However, given that we obtained complete nutrition and anthropometric data for approximately 80% of all children in the sample for 55 of the 60 countries that conducted the standard DHS surveys in the past 10 years (data from Afghanistan, Philippines, Jordan, Indonesia, and Madagascar were missing), any bias is expected to be small.

## Conclusions

The results of this cross-sectional study suggest that the current standard of measuring child undernutrition should include diet- and food-based measures because anthropometry alone failed to identify many children who have insufficient dietary intake. The findings further suggest that, like anthropometric failure, dietary diversity and micronutrient intake should be considered when assessing the global nutritional status of children.

## Notes

Supplement. **eTable 1.** Estimated Prevalence of Children Within DAF Type in Our Sample of 6-23 Months Children in 55 Countries **eTable 2.** Prevalence of Dietary Failure in Relationship With the Prevalence of the Three Different Types of Anthropometric Failure for our Sample of 6-23 Months Children in 55 Countries **eTable 3.** Average Age in Months of Children in Respective DAF Categories **eFigure 1.** Flow Diagram Showing Exclusions, Missing Data and Final Sample Size of the Study Population **eFigure 2.** Distribution of Children in DAF Groups for Different Mean Income Per Capita Levels Across 55 Countries **eFigure 3.** Prevalence of Food Groups as Recommended by WHO per Child in the Respective DAF Categories (Global) **eFigure 4.** Prevalence of Food Groups as Recommended by WHO per Child in the Respective DAF Categories (Regional)

## References

[1] UNICEF. UNICEF-WHO–The World Bank: joint child malnutrition estimates—levels and trends—2020. March 2020. Accessed August 21, 2020. https://data.unicef.org/resources/jme-report-2020/

[2] Perkins JM, Kim R, Krishna A, McGovern M, Aguayo VM, Subramanian SV. Understanding the association between stunting and child development in low- and middle-income countries: next steps for research and intervention. Soc Sci Med. 2017;193:101-109. doi:10.1016/j.socscimed.2017.09.039 29028557

[3] Patel PC, Devaraj S. Height-income association in developing countries: evidence from 14 countries. Am J Hum Biol. 2018;30(3):e23093. doi:10.1002/ajhb.23093 29282800

[4] Sudfeld CR, McCoy DC, Danaei G, . Linear growth and child development in low- and middle-income countries: a meta-analysis. Pediatrics. 2015;135(5):e1266-e1275. doi:10.1542/peds.2014-3111 25847806

[5] Horton S, Steckel RH. How Much Have Global Problems Cost the World? A Scorecard from 1900 to 2050. Cambridge University Press; 2013.

[6] Vollmer S, Harttgen K, Kupka R, Subramanian SV. Levels and trends of childhood undernutrition by wealth and education according to a Composite Index of Anthropometric Failure: evidence from 146 Demographic and Health Surveys from 39 countries. BMJ Glob Health. 2017;2(2):e000206. doi:10.1136/bmjgh-2016-000206 29081994 PMC5656130

[7] Corsi DJ, Subramanyam MA, Subramanian SV. Commentary: measuring nutritional status of children. Int J Epidemiol. 2011;40(4):1030-1036. doi:10.1093/ije/dyr108 21724577

[8] Ruel MT, Alderman H; Maternal and Child Nutrition Study Group. Nutrition-sensitive interventions and programmes: how can they help to accelerate progress in improving maternal and child nutrition? Lancet. 2013;382(9891):536-551. doi:10.1016/S0140-6736(13)60843-0 23746780

[9] Mukhopadhyay DK, Biswas AB. Food security and anthropometric failure among tribal children in Bankura, West Bengal. Indian Pediatr. 2011;48(4):311-314. doi:10.1007/s13312-011-0057-2 21169653

[10] Perkins JM, Subramanian SV, Davey Smith G, Özaltin E. Adult height, nutrition, and population health. Nutr Rev. 2016;74(3):149-165. doi:10.1093/nutrit/nuv105 26928678 PMC4892290

[11] Mann J, Truswell AS. Essentials of Human Nutrition. *5th ed.* Oxford University Press; 2017.

[12] Perkins JM, Jayatissa R, Subramanian SV. Dietary diversity and anthropometric status and failure among infants and young children in Sri Lanka. Nutrition. 2018;55-56:76-83. doi:10.1016/j.nut.2018.03.049 29980091

[13] Beckerman-Hsu JP, Chatterjee P, Kim R, Sharma S, Subramanian SV. A typology of dietary and anthropometric measures of nutritional need among children across districts and parliamentary constituencies in India, 2016. J Glob Health. 2020;10(2):020424. doi:10.7189/jogh.10.020424 33110583 PMC7569191

[14] Joe W, Rajpal S, Kim R, . Association between anthropometric-based and food-based nutritional failure among children in India, 2015. Matern Child Nutr. 2019;15(4):e12830. doi:10.1111/mcn.12830 30989801 PMC6860073

[15] Jones AD, Ickes SB, Smith LE, . World Health Organization infant and young child feeding indicators and their associations with child anthropometry: a synthesis of recent findings. Matern Child Nutr. 2014;10(1):1-17. doi:10.1111/mcn.12070 23945347 PMC6860255

[16] United Nations. United Nations world population prospects 2019. Accessed September 1, 2020. https://population.un.org/wpp/Download/Standard/Population/

[17] World Health Organization. Global Nutrition Monitoring Framework: Operational Guidance for Tracking Progress in Meeting Targets for 2025. World Health Organization; 2017.

[18] Croft TN, Marshall AM, Allen CK, Arnold F, Assaf S, Balian S. Guide to DHS Statistics. ICF; 2018.

[19] Moursi MM, Arimond M, Dewey KG, Trèche S, Ruel MT, Delpeuch F. Dietary diversity is a good predictor of the micronutrient density of the diet of 6- to 23-month-old children in Madagascar. J Nutr. 2008;138(12):2448-2453. doi:10.3945/jn.108.093971 19022971

[20] Wondafrash M, Huybregts L, Lachat C, Bouckaert KP, Kolsteren P. Dietary diversity predicts dietary quality regardless of season in 6-12-month-old infants in south-west Ethiopia. Public Health Nutr. 2016;19(14):2485-2494. doi:10.1017/S1368980016000525 27041122 PMC10270903

[21] Steyn NP, Nel JH, Nantel G, Kennedy G, Labadarios D. Food variety and dietary diversity scores in children: are they good indicators of dietary adequacy? Public Health Nutr. 2006;9(5):644-650. doi:10.1079/PHN2005912 16923296

[22] WHO, UNICEF, USAID, AED, UCDAVIS, IFPRI. Indicators for Assessing Infant and Young Child Feeding Practices: Part 1: Definitions. World Health Organization; 2008.

