# Supplementary material for: Assessment of Undernutrition Among Children in 55 Low- and Middle-Income Countries Using Dietary and Anthropometric Measures
Source: JAMA Netw Open. 2021 Aug 12;4(8):e2120627. doi: 10.1001/jamanetworkopen.2021.20627 (PMC12549096; doi:10.1001/jamanetworkopen.2021.20627)
Supplement: Supplement. — eTable 1. Estimated Prevalence of Children Within DAF Type in Our Sample of 6-23 Months Children in 55 Countries eTable 2. Prevalence of Dietary Failure in Relationship With the Prevalence of the Three Different Types of Anthropometric Failure for our Sample of 6-23 Months Children in 55 Countries eTable 3. Average Age in Months of Children in Respective DAF Categories eFigure 1. Flow Diagram Showing Exclusions, Missing Data and Final Sample Size of the Study Population eFigure 2. Distribution of Children in DAF Groups for Different Mean Income Per Capita Levels Across 55 Countries eFigure 3. Prevalence of Food Groups as Recommended by WHO per Child in the Respective DAF Categories (Global) eFigure 4. Prevalence of Food Groups as Recommended by WHO per Child in the Respective DAF Categories (Regional) [file jamanetwopen-e2120627-s001.pdf]

Supplementary Online Content

Heemann M, Kim R, Vollmer S, Subramanian SV. Assessment of undernutrition among children in 55 low- and middle-income countries using dietary and anthropometric measures. *JAMA Netw Open*. 2021;4(8):e2120627. doi:10.1001/jamanetworkopen.2021.20627

eTable 1. Estimated Prevalence of Children Within DAF Type in Our Sample of 6-23 Months Children in 55 Countries

eTable 2. Prevalence of Dietary Failure in Relationship With the Prevalence of the Three Different Types of Anthropometric Failure for our Sample of 6-23 Months Children in 55 Countries

eTable 3. Average Age in Months of Children in Respective DAF Categories

eFigure 1. Flow Diagram Showing Exclusions, Missing Data and Final Sample Size of the Study Population

eFigure 2. Distribution of Children in DAF Groups for Different Mean Income Per Capita Levels Across 55 Countries

eFigure 3. Prevalence of Food Groups as Recommended by WHO per Child in the Respective DAF Categories (Global)

eFigure 4. Prevalence of Food Groups as Recommended by WHO per Child in the Respective DAF Categories (Regional)

This supplementary material has been provided by the authors to give readers additional information about their work.

© 2021 Heemann M et al. *JAMA Network Open.*

eTable 1. Estimated Prevalence of Children Within DAF Type in Our Sample of 6-23 Months Children in 55 Countries

DAF Category	Sample Size	Percentage*	95% Conf. Interval
Both Failures	55,194	26.4%	[26.2%;26.6%]
Dietary Failure Only	67,670	45.9%	[45.6%;46.1%]
Anthropometric Failure Only	14,432	8.1%	[7.9%;8.2%]
Neither Failure	25,293	19.7%	[19.5%;19.9%]
Total	162,589		

*Equal weighting for each country

© 2021 Heemann M et al. *JAMA Network Open.*

eTable 2. Prevalence of Dietary Failure in Relationship With the Prevalence of the Three Different Types of Anthropometric Failure for our Sample of 6-23 Months Children in 55 Countries

		Wasting		Stunting		Underweight	
		Presence	Absence	Presence	Absence	Presence	Absence
**Dietary Failure**	Presence	20,094	102,770	39,372	83,492	30,061	92,803
		12.4%	63.2%	24.2%	51.3%	18.5%	57.1%
	Absence	4,146	35,579	11,134	28,591	6,498	33,227
		2.6%	21.9%	6.9%	17.6%	4.0%	20.4%
**Total**		24,240	138,349	50,506	112,083	36,559	126,030
		14.9%	85.1%	31.1%	68.9%	22.5%	77.5%
**Discordance**		65.8%		58.2%		61.1%	

Numbers are sample size and percentages weighted by country size

© 2021 Heemann M et al. *JAMA Network Open.*

eTable 3. Average Age in Months of Children in Respective DAF Categories

	**Both Failures**	**Dietary Failure Only**	**Anthropometric Failure Only**	**Neither Failure**
**Africa**	14.8	13.1	*15.7*	14.6
**Asia**	14.3	13.0	*16.6*	15.8
**Europe**	13.2	13.3	*15.6*	15.4
**South America**	14.6	13.1	*16.0*	14.8

The Italic values are the oldest average age of DAF categories per region, the underscored values are the lowest average age of DAF categories per region

© 2021 Heemann M et al. *JAMA Network Open.*

eFigure 1. Flow Diagram Showing Exclusions, Missing Data and Final Sample Size of the Study Population

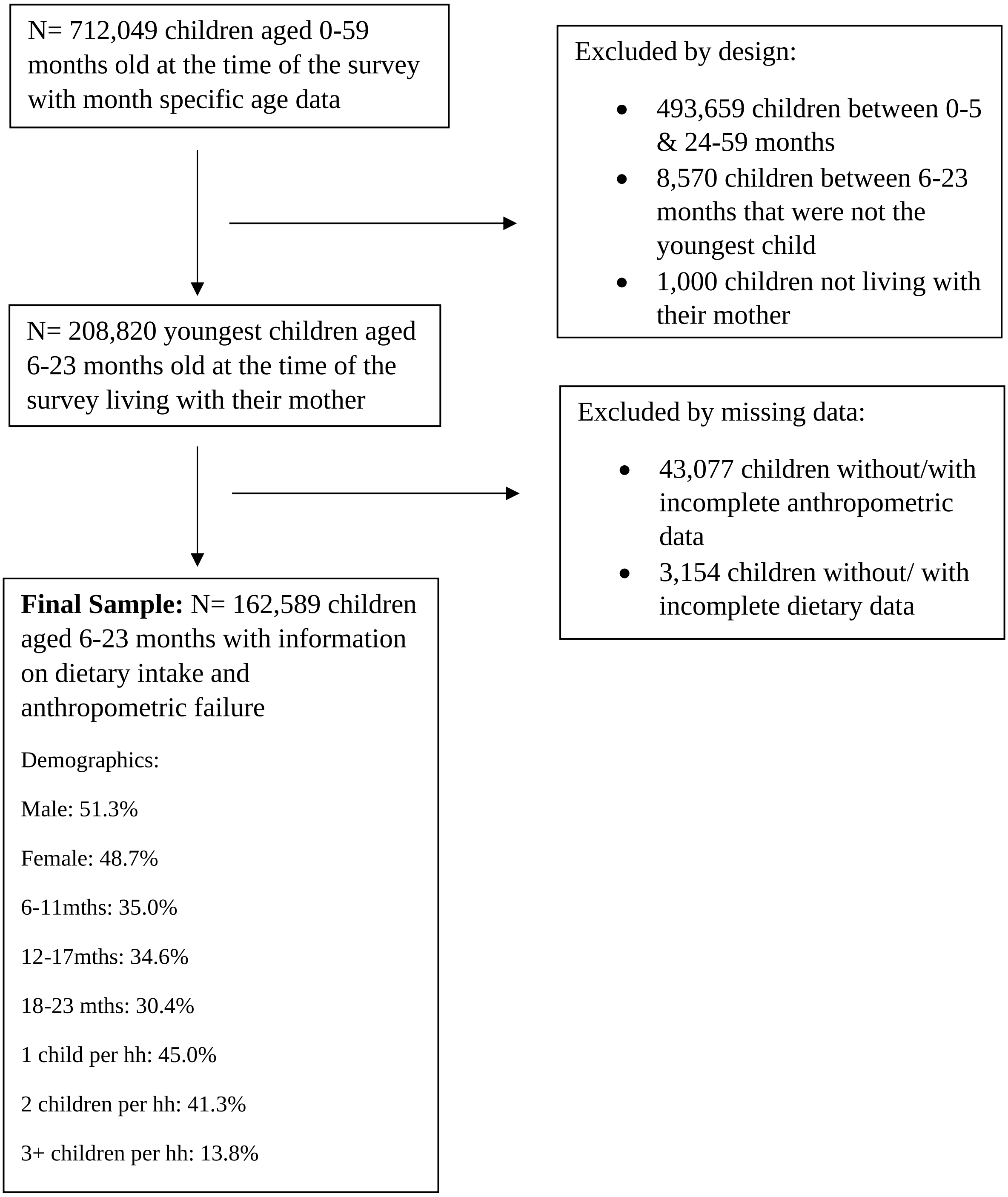

© 2021 Heemann M et al. *JAMA Network Open.*

eFigure 2. Distribution of Children in DAF Groups for Different Mean Income Per Capita Levels Across 55 Countries

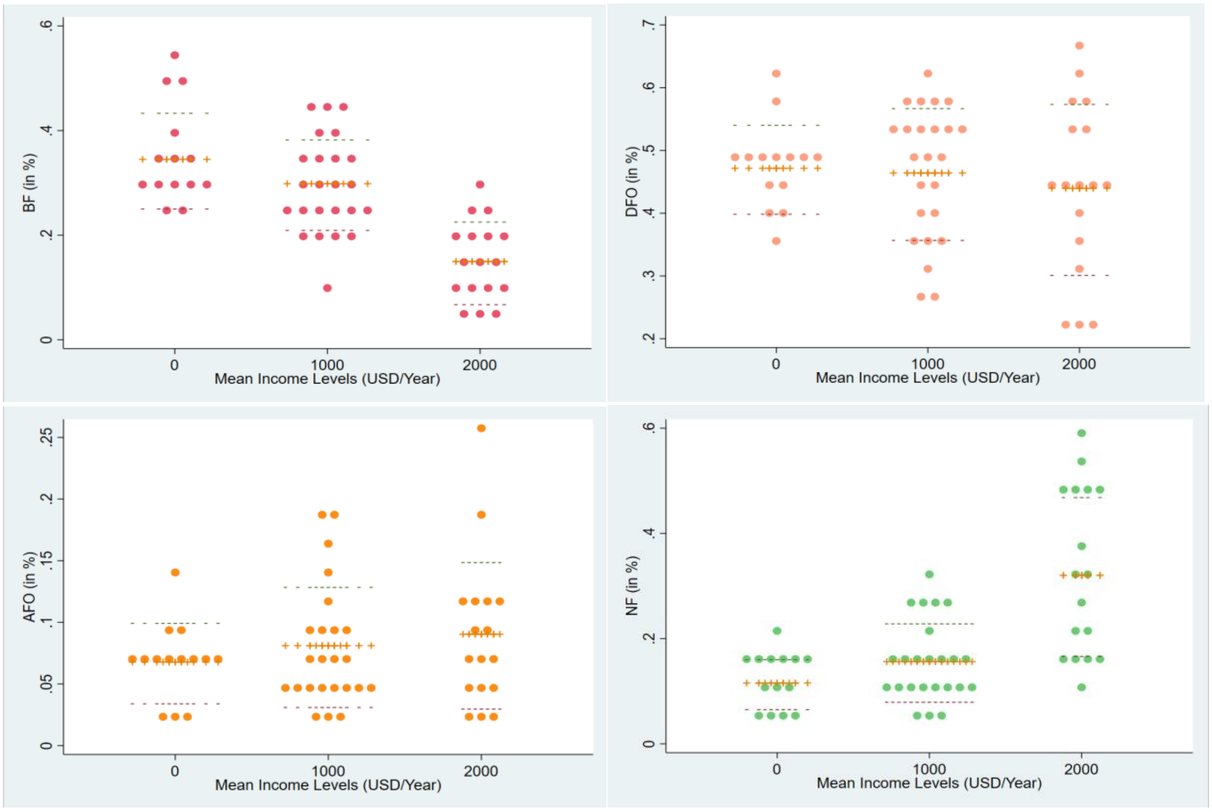

DAF categories: Dietary Failure Only (DFO), Anthropometric Failure Only (AFO), Both Failures (BF), and Neither Failure (NF). The colored dots indicate survey results for individual nations in the data set, the pluses the mean value for each Income Group and the grey dotted lines indicate the range of the mean plus and minus the standard deviation.

© 2021 Heemann M et al. *JAMA Network Open.*

eFigure 3. Prevalence of Food Groups as Recommended by WHO per Child in the Respective DAF Categories (Global)

DAF Category	Grains, roots and tubers	Legumes and nuts	Dairy products	Flesh foods	Eggs	Vitamin A rich foods	Vege-tables	Breastmilk
AFO	97.9%	53.2%	68.1%	58.9%	52.8%	86.5%	70.6%	83.8%
BF	69.7%	10.5%	32.6%	13.7%	7.3%	31.3%	10.3%	81.4%
DFO	70.1%	10.5%	34.8%	18.5%	9.3%	29.3%	12.2%	78.2%
NF	98.0%	50.8%	73.1%	66.2%	55.0%	82.4%	67.6%	77.3%

DAF categories: Dietary Failure Only (DFO), Anthropometric Failure Only (AFO), Both Failures (BF), and Neither Failure (NF). The shadings categorize average consumption levels different intensity categories, ranging from green for low levels to red for the higher levels.

eFigure 4. Prevalence of Food Groups as Recommended by WHO per Child in the Respective DAF Categories (Regional)

region	DAF Category	Grains, roots and tubers	Legumes and nuts	Dairy products	Flesh foods	Eggs	Vitamin A rich foods	Vege-tables	Breastmilk
Africa	AFO	97.3%	60.3%	56.3%	77.1%	45.5%	85.0%	66.2%	79.5%
	BF	72.5%	17.0%	18.8%	24.2%	6.9%	37.9%	9.1%	78.6%
	DFO	71.5%	14.5%	24.1%	27.2%	7.7%	34.7%	10.8%	77.2%
	NF	97.6%	55.9%	66.6%	74.7%	49.2%	83.5%	63.2%	75.7%
Asia	AFO	98.2%	47.0%	77.3%	44.2%	56.9%	88.8%	74.1%	87.8%
	BF	67.5%	5.3%	42.8%	5.8%	7.4%	26.6%	10.9%	83.7%
	DFO	67.8%	5.1%	45.9%	7.6%	10.5%	23.2%	13.1%	80.6%
	NF	98.2%	43.1%	79.9%	52.0%	60.0%	83.0%	72.7%	82.3%
Europe	AFO	97.8%	41.2%	96.8%	74.5%	69.5%	79.8%	76.3%	45.6%
	BF	71.8%	6.2%	66.2%	21.5%	17.1%	20.2%	49.2%	46.1%
	DFO	76.1%	1.8%	63.6%	21.3%	16.1%	28.9%	46.3%	48.2%
	NF	98.4%	39.7%	94.5%	81.2%	58.9%	80.1%	88.4%	51.2%
South America	AFO	98.7%	64.4%	64.3%	70.1%	69.2%	73.4%	69.1%	74.9%
	BF	80.6%	32.9%	31.9%	26.2%	21.2%	27.3%	20.3%	64.6%
	DFO	81.6%	24.6%	52.3%	30.5%	18.4%	27.9%	20.9%	56.6%
	NF	98.9%	55.9%	78.3%	77.8%	63.9%	75.5%	69.1%	67.2%

DAF categories: Dietary Failure Only (DFO), Anthropometric Failure Only (AFO), Both Failures (BF), and Neither Failure (NF). The shadings categorize average consumption levels different intensity categories, ranging from green for low levels to red for the higher levels.

© 2021 Heemann M et al. *JAMA Network Open.*
